# Professional and service-user perspectives regarding the future of mental healthcare in Israel

**DOI:** 10.1186/s13584-025-00710-7

**Published:** 2025-08-07

**Authors:** Amit Kramer, Anat Brunstein-Klomek, Nili Neuthal, Tal Nakash Bar, Dana Tzur Bitan

**Affiliations:** 1 Department of Community Mental Health, Faculty of Social Welfare and Health Sciences, University of Haifa, Mount Carmel, Haifa, 31905 Israel; 2 Shalvata Mental Health Center, Hod Hasharon, Israel; 3 Baruch Ivcher School of Psychology, Reichman University, Hertzelia, Israel

**Keywords:** Public mental health, Innovation challenges, Service users, Professionals, Attitudes

## Abstract

**Background:**

A recent call has been made in Israel to address the needs of citizens inflicted by multiple adversities such world pandemic, internal conflicts, events of mass trauma and ongoing war. Nonetheless, public’s and mental health professional’s view regarding these needs and their prioritization is not well understood. This study aims to bridge this gap in knowledge, by assessing mental health service users and professionals’ views regarding the future of mental health in Israel.

**Methods:**

Two surveys were distributed to mental health professionals and service users. Responders (286 professionals and 522 service users) were asked about their vision for future mental health services and their views regarding the integration of technology and innovation, using a close-ended measure developed by mental health professionals to address local challenges.

**Results:**

The top-rated category among professionals and service users was improving accessibility, with 75% of the service users and 82% of the professionals ranking this item as one of top-three items. Individuals with less experience with the mental healthcare system tended to rank personalized care as second-ranked priority (OR = 0.48, *p* = 0.04), whereas highly experienced individuals were more likely to rank alternatives to psychiatric hospitalization as third-ranked priority (OR = 2.99, *p* < 0.001). Professionals ranked the level of innovation in Israel’s mental healthcare as 3.37 (SD = 1.82), and service users ranked 3.18 (SD = 2.12) out of 10. Among mental health professionals, lack of resources was rated as the predominant challenge in implementing technology and innovation.

**Conclusions:**

Policy and decision-makers in Israel should consider addressing the issue of mental healthcare accessibility as top priority when planning a mental health reform, as well as routes to improve personalized care and alternative to psychiatric admissions. Steps should be taken to improve innovation and technology as means to improve the quality of mental healthcare in Israel.

**Supplementary Information:**

The online version contains supplementary material available at 10.1186/s13584-025-00710-7.

Many countries worldwide have recently initiated comprehensive mental health reforms to address the increasing demand for mental health services. To illustrate, Denmark has launched a 10-year plan to prioritize prevention and early intervention [1, 2]; the National Health Service (NHS) in England has expanded the ‘Improving Access to Psychological Therapies (IAPT)’ program to provide evidence-based psychological treatments [3]; in Australia, the government expanded its existing mental health framework to provide mental health services to young people [4] in a stepped care model. These efforts clearly reflect the growing recognition that mental health care needs to evolve beyond conventional models to meet current needs.

Building on this shared recognition, different countries have initiated distinct reforms aimed to address each country’s unique needs and challenges. For instance, Italy has adopted the Trieste model to meet the need for deinstitutionalization [5, 6]. In the UK, accessibility to mental health services has been a significant challenge, particularly for at-risk populations [3, 7, 8]. Data indicating that young people have the worst access to mental health services in Australia has led to the creation of ‘Headspace’, a nationwide initiative targeting early intervention for young people [9]. Many countries worldwide have acknowledged the increasing distress caused the COVID-19 pandemic and called for appropriate means to address it [10, 11]. Thus, to plan and initiate a mental health reform in Israel, one needs to first evaluate the unique needs of service users, as well as the challenges of professionals aiming to address them.

Although many studies provide evidence of the growing demand for mental health services in Israel [12–17], the specific needs, and the challenges of implementing innovative solutions to address them, have yet to be clearly defined. As many countries worldwide integrate technology and use of digital mental healthcare as means to overcome systemic challenges [18], additional information is needed to understand potential barriers for the use of these innovative technologies in Israel [19]. As recently acknowledged by the world health organization (WHO), such evaluation may enable to overcome the major impediments for accessing new digital health tools, and improve quality and accessibility of care [20]. Previous studies have further demonstrated that different populations and professional sectors report different challenges for implementation of innovative interventions [21], and that personalizing solutions to meet people’s unique needs may improve mental health outcomes [22]. Thus, additional research is needed to evaluate the unique needs and challenges of service users and professionals, as well as the unique characteristics of those expressing specific needs.

The present study aims to bridge these gaps in knowledge, by exploring service-users and mental health professionals’ perceptions regarding the nature of change required in mental health services in Israel. This exploration was aimed to provide an empirical foundation to inform policy change in Israel, as part of a strategy planning initiated by the National Institute for Healthcare Policy (NIHP). As the Israeli mental healthcare struggles with continuous transition of professionals’ to the private sector [23], we aimed to focus on both service users and mental healthcare professionals. Specifically, we explored (1) individuals’ attitudes towards the future of mental health care, among service-users and mental health professionals, (2) the potential role of technology and innovation in addressing current needs from service-users’ perspective, (3) the perceived challenges in the implementation of technology and innovation in the public mental health system from professionals’ point-of-view, (4) the unique characteristics of service users and professionals who report of these challenges.

## Methods

### Participants

Service users survey was distributed in social media and mailing lists of individuals who self-identify as mental healthcare users (such as Facebook pages of individual with lived experience), as well as in broader social media platforms of non-professionals. The inclusion criteria for the service-users were providing informed consent and a sufficient understanding of Hebrew. Participants had to be adults and above the age of 18 to be eligible to complete the survey, and could join the study regardless of their citizenship or their previous utilization of public mental health services. Professionals were recruited via social media designated for professionals (WhatsApp, Facebook) and through professionals’ mental healthcare mailing lists. The providers were requested to indicate their profession upon entering the survey. The inclusion criteria for professionals were being a mental healthcare provider and a sufficient understanding of Hebrew. Providers could be from any professional background, either currently or previously employed in public mental healthcare settings.

Overall, 1,316 subjects responded to the two surveys, with 761 individuals responding to the survey in the service-users and 555 individuals in the professional’s sample. From the service-user data, six participants (0.7%) did not sign the informed consent, and 239 (31.4%) signed consent but did not start the survey, thus resulting in a total of 522 participants (68.6%). In the professional survey, 14 therapists (2.5%) did not consent to participate, and 256 therapists (33.64%) signed consent but did not start the survey, thus resulting in a total of 286 (51.5%). Total analyzed sample included 808 participants overall, with 522 participants from the service-users and 286 professionals.

### Procedure

The study received the approval of the Institutional Review Board (IRB) at Richmann University (reference number: P_2024080). An online survey was distributed during April-May 2024. The online survey was administered through the Qualtrics platform. Participants were asked to electronically sign an informed consent online. After agreeing to participate, participants completed a demographic questionnaire followed by assessments of the study variables.

### Measures

Two short surveys were developed to assess the current needs and challenges of mental health in Israel. The service-users survey included twelve items, with four pertaining to demographics, five pertaining to clinical characteristics (such as previous admissions or use of rehabilitation service), and three pertaining to vision and innovation. The professional’s survey included eleven items, with four pertaining to demographics, four to professional characteristics (such as professional training, years of experience, whether they are working in the public mental health), and three items for future vision and innovation.

#### Public mental health vision– service users and professionals (one item)

Participants in both samples were asked to choose the top five priorities for the future of mental health in Israel from list of 12 options: personal-tailored treatments, monitoring treatment effectiveness, adding innovative treatment options (e.g. virtual reality), mutual decision making, reinforcement of availability and accessibility, reduction of psychiatric medication, resilience training, promoting alternatives for inpatient care, integrating mental treatment in primary care and emergency rooms, adding rehabilitation and community services and improving treatment continuity. The following instruction was given: “You have been chosen to be part of a team working on formulating the vision for mental health in Israel for the year 2034. Please choose the five topics you believe are the most important to promote as part of this vision”. These items were identified by senior mental health professionals participating in a summit of the Israel National Institute for Health Policy Research (NIHP, May 2024).

#### Mental health technology and innovation needs– service users (two items)

Participants in the service-users were also requested to rate the level of innovation in the mental health system in Israel on a 10-point Likert scale ranging from 1 (very poor) to 10 (very advanced). Next, they were asked to choose the three main topics that require innovative and technological attention out of a total of nine options which were similarly developed by senior mental health professionals: improving accessibility and availability, improving accessibility to well-established knowledge on mental health, online remote therapy, digital tools for mental health management, digital tools for self-monitoring of mental health state, digital tools for self-treatment, and digital tools for self-diagnosis or other. The following instruction was given: “Select the three main areas in which technology needs to be integrated into the field of mental health”.

#### Mental health technology and innovation implementation challenges - professionals (two items)

The professionals were asked to mark the three greatest challenges in implementation of technology and innovation in mental health care. The following eight options were displayed: privacy and information security, difficulties to create change and collaborate with therapists, objections and lack of cooperation from patients, difficulties in development and application of technology tools suited to the patients’ and therapists’ needs, fear of harming the therapeutic alliance and therapeutic quality, difficulties in accessibility to technology among specific populations, lack of resources, organizational culture and employee burnout, or other. The following instruction was given: “Please mark the three main challenges in promoting innovative thinking and technology in the mental health system”.

### Statistical analysis

Descriptive statistics were reported using means and standard deviations. The predictive effects of service-users and professionals’ characteristics were evaluated using hierarchical binary logistic regressions for two outcome variables: the likelihood of rating one of three main priorities in future vision of mental health and the same for technology and innovation. In these regressions, demographics were entered in Block 1 (including gender, age, and family status), and clinical or professional variables (such as familiarity with public mental health system, past treatment, and past hospitalization for service users, and professional training, years of experience, working in public services for the professional sample) in Block 2. Multicollinearity assumption was explored and verified for all analyses (VIF < 2.5) [24]. Statistical analysis was preformed using SPSS software, version 27.0 (SPSS, Chicago, IL, U.S.A).

## Results

### Professionals and service users sample characteristics

Table 1 presents the demographic, clinical and professional characteristics of the two study samples. For the *service-users*, the majority were between the age of 40–59 (46.74%), primarily females (75.86%) and living in central Israel (66.48%). Less than half of the sample (45.22%) were either single or divorced, and 42.34% were married. The majority of the sample reported that they have previously received public mental health treatment (59.96%), and 42.72% reported receiving social benefit or rehabilitation support. 61.69% reported that a family member has been treated in mental health public care. In the mental health *professional sample*, 31.69% of the participants were between the ages 40–49 and 27.82% were under the age of 39. The sample mostly included females (81.12%) from central Israel (77.62%). Most of the sample reported being married (77.27%). In terms of professional qualification, 28.32% were psychologists, 23.08% were social workers, 16.79% were occupational or art therapists, and 16.08% were psychiatrists. Most of the participants had above 20 years of experience (33.57%), with 83% currently employed in the public mental health sector.

**Table 1:** Service-users and professionals’ demographic, clinical and professional characteristics

Service-users (*N* = 522)				Therapists (*N* = 286)		
	N	%			N	%
Age: 0–29	108	20.69		Age: 0–29	79	27.82
30–39	134	25.67		40–49 years	90	31.69
40–59	244	46.74		50–59 years	78	27.46
Above 60	36	6.90		Above 60 years	37	13.03
Sex female	396	75.86		Sex female	232	81.12
Male	118	22.61		Male	52	18.18
Other	8	1.53		Other	1	0.35
Family status				Family Status		
Single	159	30.46		Single	23	8.04
In a relationship	55	10.54		In a relationship	20	6.99
Married/other	221	42.34		Married/other	221	77.27
Divorce/separated	77	14.76		Divorced/separated	20	6.99
Widowed	10	1.92		Profession		
Past mental health treatment				Psychiatrist	46	16.08
Yes	313	59.96		Psychologist	81	28.32
No	206	39.46		Social worker	66	23.08
Family member in public setting				Occupational/art therapist	48	16.79
Yes	322	61.69		Other	45	15.73
No	200	38.31		Years of Experience		
Familiarity with public setting				Under 5 years	47	16.43
Low	65	12.45		5–10 years	68	23.78
Medium	191	36.59		10–20 years	75	26.22
High	266	50.96		Above 20 years	96	33.57
Social benefit/rehabilitation service				Working in public sector	238	83.22
Yes	223	42.72		No	16	5.59
No	296	56.7		Not now, but in the past	32	11.19
				Familiarity with public settings		
				Low	14	4.9
				Medium	101	35.31
				High	170	59.44

### Mental health vision, technology and innovation among service users

Table 2 presents the rankings of the service users’ sample for the future of mental health care, and innovation and technology. Service users rated the current state of technology and innovation in public mental health as 3.18 (SD = 2.12) out of 10. The top-rated three categories for the vision of mental health care were (1) improving availability and accessibility (75.47%), (2) personalization of therapy (60.15%), and (3) creating alternatives to psychiatric hospitalization (53.44%). The top-rated three categories for innovation and technology were (1) improving accessibility and availability (69.55%), (2) improving opportunities for online remote therapy (48.27%) and (3) improving accessibility to knowledge on mental health (45.02%).

**Table 2:** Means, sds, and frequencies of service-user’s perceptions (*N* = 522)

Variable	
*Service-users main vision for the future of mental health care (N*,* %)* ^*a*^	
Improving availability and accessibility	394 (75.47)
Personalization of therapy	314 (60.15)
Alternatives to psychiatric hospitalization	279 (53.44)
Expending rehabilitation and community services	248 (47.51)
Resilience training	194 (37.16)
Mutual patient-therapist decision-making	190 (36.40)
Improving treatment continuity	181 (34.67)
Reducing use of psychiatric medications	174 (33.33)
Dissemination of innovative treatment methods	156 (29.88)
Integration of mental healthcare in primary care and emergency rooms	155 (29.70)
Familial involvement in treatment	122 (23.37)
Other	69 (13.21)
*Service-users main fields requiring innovation and technology (N*,* %)* ^*b*^	
Improving accessibility and availability	363 (69.55)
Online remote therapy	252 (48.27)
Improving accessibility to well-established knowledge on mental health	235 (45.02)
Digital tools for mental health management	200 (38.31)
Digital tools for self-monitoring of mental health state	175 (33.52)
Digital tools for self-treatment	111 (21.26)
Digital tools for self-diagnosis	71 (13.06)
Other	36 (6.90)
*Service-users’ ratings of technology and innovation in mental health services (M*,* SD)* ^*c*^	3.18 (2.12)

*Notes*. ^a^ Participants were requested to mark five primary visions for the future of mental healthcare of a list of 12 suggested visions. ^b^ Participants were asked to choose three main fields for innovation and technology from a list of eight options. ^c^ Rated on a scale of 1 (*not innovative at all*) to 10 (*very innovative*)

### Mental health vision, technology and innovation challenges among professionals

Table 3 presents the rankings of the professionals’ sample for the future vision of mental health care, and perceived challenges of integrating technology and innovation in the field. Professionals rated the current state of technology and innovation in public mental health as 3.37 (SD = 1.82) out of 10. The three main priorities for the future of mental health reported by professionals were (1) improving availability and accessibility (82.52%), (2) expanding rehabilitation and community services (59.09%) and (3) improving treatment continuity (56.29%). In addition, professionals stated that the three main challenges in implementing innovation and technology are (1) lack of resources in the public mental health sector (59.79%), (2) the existing organizational culture (37.06), and (3) employee burnout (36.36%).

**Table 3:** Means, sds, and frequencies of professionals’ perceptions (*N* = 286)

Variable	
*Professionals main vision for the future of mental health care (N*,* %)* ^*a*^	
Improving availability and accessibility	236 (82.52)
Expanding rehabilitation and community services	169 (59.09)
Improving treatment continuity	161 (56.29)
Personalization of therapy	141 (49.3)
Alternatives to psychiatric hospitalization	119 (41.61)
Familial involvement in treatment	99 (34.62)
Integration of mental healthcare in primary care and emergency rooms	94 (32.87)
Resilience training	80 (27.97)
Mutual patient-therapist decision making	65 (22.73)
Monitoring treatment effectiveness	64 (22.38)
Adding innovative treatment methods	50 (17.48)
Dissemination of innovative treatment methods	39 (13.64)
Other	31 (10.84)
*Professionals reported challenges of integrating innovation and technology (N*,* %)* ^*b*^	
Lack of resources	171 (59.79)
Organizational culture	106 (37.06)
Employee burnout	104 (36.36)
Difficulties to create change and collaborate with therapists	95 (33.22)
Fear of harming the therapy alliance and quality	94 (32.87)
Difficulties in accessibility to technology among specific populations	82 (29.02)
Technology tools suitability to patients’ and therapists’ needs	78 (27.27)
Privacy and information security issues	77 (26.92)
Objections and lack of cooperation from patients	32 (11.19)
*Professionals’ ratings of technology and innovation in mental health services (M*,* SD)* ^*c*^	3.37 (1.82)

*Notes*. ^a^ Participants were requested to mark five primary visions for the future of mental healthcare of a list of 12 suggested visions. ^b^ Participants were asked to choose three main fields for innovation and technology from a list of nine options. ^c^ Rated on a scale of 1 (*not innovative at all*) to 10 (*very innovative*)

### Predictive characteristics of top-three needs and challenges among service users

The predictive role of clinical and demographic characteristics on mental health needs and challenges was assessed through a multivariate hierarchical logistic regression. Full models are elaborated in the Supplementary Materials (Tables S1 to S6).

For the service users’ sample (see Table 4), females were those most likely to report accessibility needs as a top priority (OR = 2.07, 95% CI = 1.31–3.26, *p* = 0.002), as well as the primary area needing attention through technology (OR = 1.65, 95% CI = 1.06–2.56, *p* = 0.024). People above the age of 40 (OR = 1.73, 95% CI = 1.00-2.97, *p* = 0.046), and people who were previously hospitalized (OR = 1.84, 95% CI = 1.03–3.29, *p* = 0.039) also ranked accessibility as top priority of innovation. Personalized treatment as second-ranked priority was most prevalent among singles (OR = 0.45, 95% CI = 0.27–0.74, *p* < 0.001; OR = 0.47, 95% CI = 0.24–0.91, *p* = 0.03), people with less familiarity with mental health system (OR = 0.48, 95% CI = 0.24–0.98, *p* = 0.04; OR = 0.47, 95% CI = 0.23–0.95, *p* = 0.04) and people who do not receive rehabilitation services (OR = 0.46, 95% CI = 0.25–0.86, *p* = 0.02). Alternatives to psychiatric hospitalizations was ranked third mostly among individuals with medium (OR = 2.31, 95% CI = 1.20–4.44, *p* = 0.01) or high (OR = 2.99, 95% CI = 1.55–5.77, *p* < 0.001) familiarity with mental health system. Improving access to knowledge was the third-ranked area requiring technological advancement among individuals highly familiar with the system (OR = 2.21, 95% CI = 1.18–4.13, *p* = 0.01).

**Table 4:** Predictors of top three priorities for mental health vision and use of technology among the service-users

	Top ranked vision				Top ranked challenge to technology and innovation			
**First ranked**	Accessibility				Accessibility			
	B	OR	95% CI	*p*	B	OR	95% CI	*p*
Gender	**0.72**	**2.07**	**1.31–3.26**	**0.002**	**0.50**	**1.65**	**1.06–2.56**	**0.024**
Age: 40 and above	-0.23	0.79	0.42–1.47	0.46	**0.55**	**1.73**	**1.00-2.97**	**0.046**
Past psychiatric hospitalization	0.32	1.37	0.73–2.57	0.31	**0.61**	**1.84**	**1.03–3.29**	**0.039**
**Second ranked**	Personalized treatment				Online Remote Therapy			
Family status: Married/relationship	**-0.79**	**0.45**	**0.27–0.74**	**0.00**	0.11	1.11	0.70–1.76	0.64
Family status: Separated/divorced	**-0.75**	**0.47**	**0.24–0.91**	**0.03**	0.36	1.44	0.77–2.70	0.25
Medium familiarity with public mental health	**-0.71**	**0.48**	**0.24–0.98**	**0.04**	-0.32	0.72	0.38–1.36	0.31
High familiarity with public mental health	**-0.74**	**0.47**	**0.23–0.95**	**0.04**	-0.55	0.57	0.30–1.08	0.09
Social benefit/rehabilitation service	**-0.76**	**0.46**	**0.25–0.86**	**0.02**	0.18	1.19	0.67–2.12	0.55
**Third ranked**	Alternatives to psychiatric hospitalization				Improving accessibility to knowledge			
Medium familiarity with public mental health	**0.83**	**2.31**	**1.20–4.44**	**0.01**	0.42	1.53	0.82–2.87	0.18
High familiarity with public mental health	**1.10**	**2.99**	**1.55–5.77**	**0.00**	**0.80**	**2.21**	**1.18–4.13**	**0.01**

*Notes.* Table presents only significant predictors of each ranked need and challenge. Full models are elaborated in the Supplementary Materials. Reference group for age (0–29 years), gender (male), family status (single), familiarity (low), past hospitalization (no), social benefit/rehabilitation service (no)

### Predictive characteristics of top-three needs and challenges among professionals

Among professionals (see Table 5), accessibility was identified as the top priority for the future of mental health services. Psychiatrists were more likely to rate this factor as top priority compared to art and occupational therapists (OR = 0.20, 95%CI = 0.06–0.65, *p* = 0.01). Organizational culture was reported as the second-ranked challenge for the implementation of new technologies, and was reported mostly by professionals of younger age compared to professionals over the age of 50 (OR = 0.46, 95% CI = 0.24–0.87, *p* = 0.02), and by psychologists and social workers compared to psychiatrists (OR = 2.48, 95% CI = 1.11–5.55, *p* = 0.03). Improving treatment continuity as third-ranked need was more likely to be reported by professionals aged 40–49 (OR = 0.47, 95% CI = 0.24–0.93, *p* = 0.03) compared to younger professionals.

**Table 5:** Predictors of top three priorities for mental health vision and challenges in the integration of technology among professionals

	Top ranked vision				Top ranked challenge to technology and innovation			
**First ranked**	Accessibility				Lack of resources			
	B	OR	95% CI	*p*	B	OR	95% CI	*p*
Art and occupational therapists, other	**-1.63**	**0.2**	**0.06–0.65**	**0.01**	0.33	1.4	0.63–3.1	0.41
**Second ranked**	Expanding rehabilitation community services				Organizational culture			
Age: 50 and above	-0.38	0.68	0.36–1.27	0.23	**-0.76**	**0.46**	**0.24–0.87**	**0.02**
Psychologists and social workers	-0.25	0.77	0.36–1.60	0.49	**0.91**	**2.48**	**1.11–5.55**	**0.03**
**Third ranked**	Improving treatment continuity				Employee burnout			
Age: 40–49	**-0.74**	**0.47**	**0.24–0.93**	**0.03**	-0.47	0.62	0.31–1.23	0.17

*Notes.* Table presents only significant predictors of each ranked need and challenge. Full models are elaborated in the Supplementary Materials. Reference group for age (0–29 years), profession (psychiatrics)

## Discussion

The current study was aimed to assess service-users’ and mental health professionals’ perceptions about the vision of mental health services, and the role of technology and innovation. Across both samples, the issue of accessibility emerged as one of the top-ranked need for mental healthcare vision as well as a target for technology and innovation. Other top-ranked needs expressed by service users included the need to provide personalized care and the development of alternatives to psychiatric hospitalization, as well as utilizing technology for the sake of online therapy and for psycho-educative purposes. These findings echo recent calls to shift from a hospital-biomedical -based to an integrated community-based model, which harnesses technology and family involvement as means to improve accessibility to quality-care [25]. Female service users, people of older age, and those who were previously hospitalized were those most likely to rank accessibility as top priority of innovation. Personalized treatment was ranked second, mostly by singles with no rehabilitation services, and individuals with less experience with the mental healthcare system. Alternatives to psychiatric hospitalization was ranked third mostly by individuals with high familiarity with the mental healthcare. Among professionals, accessibility was identified as the top priority mostly by psychiatrists. Younger professionals, and mostly psychologists and social workers, reported organizational culture as second-ranked challenge to innovation. The greatest challenge in implementing technology and innovation among professionals was a lack of resources. Finally, both samples rated the level of Israel’s mental health innovation as relatively low, with a mean score of 3 out of 10.

The findings of the current study highlight the importance of availability of mental health services for both service-users and professionals. Recent studies conducted in Israel demonstrate a sharp increase in PTSD symptomatology as well as in demand for mental health treatments after October 7th [23, 26]. These findings resonate with recent reports indicating a constant incline in mental health needs worldwide [27]. To illustrate, in the U.S. there has been an increase in serious psychological distress and suicide-related outcomes among young adult (18–25), as well as an increase in mood disorders among adolescents (12–17) and young adults (18–25) [28]. In a systematic review and meta-analysis conducted in Norway, a solid increase in mental health problems among young individuals and especially among females was also reported [29]. These findings suggest that this increase in mental healthcare demand is universal.

Analyses of the predictive profiles of individuals’ rankings indicates that females, people of older age, and those who were previously hospitalized were those most likely to rank accessibility as top priority of innovation, and that individuals highly familiar with the mental healthcare also prioritize the need for alternatives to psychiatric hospitalization. Studies conducted after October 7th demonstrate that women are disproportionally affected by the attack and subsequent war [30, 31] thus potentially contributing to the increased priority for availability of mental health services among this population. Other studies demonstrate significant associations between mental illness severity and perceived need of mental health services [32]. Furthermore, serious mental illness has been associated with greater use of general and mental health services [33, 34] and as target for balacing homes, which provide alternative to psychiatric admission [35]. Taken together, these results suggest that there are distinct populations in need of distinct services to address their mental health needs.

Mental health professionals perceived the lack of resources as a significant barrier to implementing technology and innovation, and ranked the state of innovation in mental healthcare in Israel as relatively poor. These findings resonate with reports worldwide regarding major gaps in resources, services and technologies for mental healthcare [27]. The persistence of the resource gap in mental healthcare is also likely to limit the implementation of innovation and technology in daily practice [36]. For example, major workload and time constraints may limit clinicians’ readiness to engage in innovative interventions implementation, and lead to burnout and reduced willingness to participate in ongoing intiatives. Addressing the resources gap is therefore likely not only to improve healthcare, but also provide professionals with opportunities for professional growth.

Several limitations should be acknowledged. The study was conducted as a self-report survey; therefore, the results are restricted to the respondents’ subjective experience. Some of the issues raised in the survey could have been beyond the scope of knowledge for some of the participants (for example, how innovative the mental health system is). This study employed a self-developed scale to evaluate the unique needs and challenges of the Israeli population. Future studies should aim to deepen the investigation regarding the implementation challenges while utilizing qualitative designs. As this study focused only on the three top ranked challenges, additional studies are needed to fully delineate the unique needs of different service-users and professionals, to inform personalized care. Although the comparison between service users and professionals was not a direct objective of the study, the results can suggest that professionals and service users have similar views regarding some of the aspects of mental healthcare functioning (such as lack of innovation and accessibility issues), but different perspectives about other aspects (such as the need for hospitalization alternatives, which was ranked higher by service-users). Additional studies are needed to examine whether these gaps in perceived needs impact mental healthcare management and how to adequately address them to meet patient’s needs.

Notwithstanding these limitations, our study indicates that issues of accessibility, personalized care and psychiatric hospitalization alternatives serve as the most prominent challenges of Israel’s mental health services. Despite institutional efforts, mental health public service accessibility remains a significant challenge reported by both service-users and professionals. This need should inform decision makers to develop strategies to improve accessibility and provide resources for technological and innovative initiatives to ease access to mental health services. Furthermore, the results demonstrate that different service users and different professionals struggle with different barriers of innovation implementation. As the era of personalized care is underway, these results highlight the need to adjust policies to these differential needs, rather than employing a ‘one size fits all’ strategy. As the mental healthcare system in Israel currently struggles with a significant reduction in mental healthcare personnel [23], the results of this study demonstrate that such personalized approach is crucial not only for service users, but also for professionals employed in the public sector and struggle to develop professionally. Such viewpoint of the mental health care might facilitate its development, and improve overall quality of care.

## Supplementary Information

Below is the link to the electronic supplementary material.

Supplementary Material 1

## Data Availability

No datasets were generated or analysed during the current study.

## Abbreviations

NHS: National health service
IAPT: Improving access to psychological therapies
NIHP: National institute for healthcare policy
IRB: Institutional review board
SD: Standard deviation
OR: Odds ratio
CI: Confidence interval
VIF: Variance inflation factor
SPSS: Statistical Package for the Social Sciences
