# Supplementary material for: Professional and service-user perspectives regarding the future of mental healthcare in Israel
Source: Isr J Health Policy Res. 2025 Aug 7;14:48. doi: 10.1186/s13584-025-00710-7 (PMC12330161; doi:10.1186/s13584-025-00710-7)
Supplement: Supplementary file 1 — Supplementary Material 1 [file 13584_2025_710_MOESM1_ESM.docx]

**Supplementary Materials**

Table S1. Full model for accessibility as top-ranked factor for mental health vision and and use of technology *among service-users*

|  | Accessibility as a top vision | | | | Accessibility in technology and innovation | | | |
| --- | --- | --- | --- | --- | --- | --- | --- | --- |
| *Demographic factors (Block 1)* | B | OR | 95% CI | *p* | B | OR | 95% CI | *p* |
| Gender | **0.72** | **2.07** | **1.31-3.26** | **0.002** | **0.50** | **1.65** | **1.06-2.56** | **0.024** |
| Age: 30-39 | -0.49 | 0.61 | 0.32-1.15 | 0.13 | 0.14 | 1.15 | 0.66-2.00 | 0.62 |
| Age: 40 and above | -0.23 | 0.79 | 0.42-1.47 | 0.46 | **0.55** | **1.73** | **1.00-2.97** | **0.046** |
| Family status: Married/relationship | 0.35 | 1.42 | 0.86-2.34 | 0.17 | 0.07 | 1.08 | 0.68-1.71 | 0.75 |
| Family status: Separated/divorced | -0.22 | 0.8 | 0.42-1.51 | 0.49 | -0.59 | 0.55 | 0.30-1.02 | 0.06 |
| *Clinical factors (Block 2)* |  |  |  |  |  |  |  |  |
| Medium familiarity with public mental health | 0.39 | 1.48 | 0.73-3.00 | 0.27 | 0.19 | 1.21 | 0.63-2.32 | 0.56 |
| High familiarity with public mental health | 0.26 | 1.265 | 0.65-2.58 | 0.46 | 0.35 | 1.42 | 0.74-2.71 | 0.29 |
| Past treatment in public service | -0.29 | 1.75 | 0.42-1.33 | 0.33 | 0.11 | 1.12 | 0.67-1.87 | 0.66 |
| Family member treated in public service | 0.21 | 1.24 | 0.80-1.93 | 0.33 | 0.20 | 1.22 | 0.81-1.83 | 0.34 |
| Past psychiatric hospitalization | 0.32 | 1.37 | 0.73-2.57 | 0.31 | **0.61** | **1.84** | **1.03-3.29** | **0.039** |
| Social benefit/rehabilitation service | 0.6 | 1.82 | 0.95-3.48 | 0.07 | 0.04 | 1.04 | 0.58-1.87 | 0.89 |

*Notes.* Reference group for age (0-29 years), gender (male), family status (single), familiarity (low), past mental health treatment in public service (no), family member treated in public mental health service (no), past hospitalization (no), national security insurance (no).

Table S2. Full model of accessibility as a top priority in vision and lack of resources as a main challenge in technology implementation *among professionals*

|  | Accessibility as a top priority vision | | | | Lack of resources as a main challenge | | | |
| --- | --- | --- | --- | --- | --- | --- | --- | --- |
| *Demographic factors (Block 1)* | B | OR | 95% CI | *p* | B | OR | 95% CI | *p* |
| Gender | 0.33 | 1.39 | 0.65-2.99 | 0.4 | -0.21 | 0.81 | 0.42-1.55 | 0.52 |
| Age: 40-49 | 0.29 | 1.33 | 0.53-3.39 | 0.54 | -0.002 | 0.1 | 0.52-1.91 | 0.1 |
| Age: 50 and above | -0.64 | 0.53 | 0.24-1.18 | 0.12 | -0.26 | 0.77 | 0.41-1.44 | 0.41 |
| Family status: Married/relationship | -0.64 | 0.53 | 0.11-2.44 | 0.41 | -0.29 | 0.75 | 0.29-1.97 | 0.56 |
| Family status: Separated/divorced | 0.18 | 1.2 | 0.14 – 10.23 | 0.87 | -0.27 | 0.77 | 0.2-2.94 | 0.7 |
| *Professional factors (Block 2)* |  |  |  |  |  |  |  |  |
| Psychologists and social workers | 0.27 | 0.77 | 0.23-2.53 | 0.66 | -0.16 | 0.85 | 0.42-1.75 | 0.66 |
| Art and occupational therapists, other | **-1.63** | **0.2** | **0.06-0.65** | **0.01** | 0.33 | 1.4 | 0.63-3.1 | 0.41 |
| Years of experience: above 10 years | 0.69 | 1.98 | 0.82-4.82 | 0.13 | -0.22 | 0.8 | 0.402-1.6 | 0.53 |
| Working in the Public Service | -0.81 | 0.45 | 0.14-1.45 | 0.18 | -0.84 | 0.43 | 0.15-1.24 | 0.12 |
| High familiarity with public mental health | -0.07 | 0.93 | 0.45-1.93 | 0.84 | 0.21 | 1.23 | 0.72-2.1 | 0.45 |

*Notes.* Reference group for age (0-39 years), gender (male), family status (single), profession (psychiatrics), years of experience (under 10 years), working in public service (no), familiarly with public mental health (low and medium).

Table S3. Full model of predictors of 'personalized treatment' as second-ranked priority in future vision, and 'integrating technology in remote treatment' as the second main technological need among *service users*

|  | Personalized treatment as a secondary priority in vision | | | | Integrating technology into remote treatment as a secondary priority | | | |
| --- | --- | --- | --- | --- | --- | --- | --- | --- |
| *Demographic factors (Block 1)* | B | OR | 95% CI | *p* | B | OR | 95% CI | *p* |
| Gender | 0.88 | 1.09 | 0.68-1.75 | 0.71 | -0.02 | 0.97 | 0.62-1.52 | 0.91 |
| Age: 30-39 | -0.12 | 0.88 | 0.48-1.63 | 0.70 | -0.24 | 0.78 | 0.44-1.37 | 0.39 |
| Age: 40 and above | 0.03 | 1.03 | 0.58-1.83 | 0.90 | 0.20 | 1.22 | 0.72-2.08 | 0.44 |
| Family status: Married/relationship | **-0.79** | **0.45** | **0.27-0.74** | **0.00** | 0.11 | 1.11 | 0.70-1.76 | 0.64 |
| Family status: Separated/divorced | **-0.75** | **0.47** | **0.24-0.91** | **0.03** | 0.36 | 1.44 | 0.77-2.70 | 0.25 |
| *Clinical factors (Block 2)* |  |  |  |  |  |  |  |  |
| Medium familiarity with public mental health | **-0.71** | **0.48** | **0.24-0.98** | **0.04** | -0.32 | 0.72 | 0.38-1.36 | 0.31 |
| High familiarity with public mental health | **-0.74** | **0.47** | **0.23-0.95** | **0.04** | -0.55 | 0.57 | 0.30-1.08 | 0.09 |
| Past treatment in public service | 0.08 | 1.07 | 0.65-1.78 | 0.76 | -0.04 | 0.95 | 0.58-1.55 | 0.85 |
| Family member treated in public service | 0.38 | 1.47 | 0.96-2.25 | 0.07 | -0.06 | 0.94 | 0.63-1.39 | 0.75 |
| Past psychiatric hospitalization | 0.06 | 1.06 | 0.56-2.00 | 0.85 | -0.26 | 0.76 | 0.43-1.36 | 0.36 |
| Social benefit/rehabilitation service | **-0.76** | **0.46** | **0.25-0.86** | **0.02** | 0.18 | 1.19 | 0.67-2.12 | 0.55 |

*Notes.* Reference group for age (0-29 years), gender (male), family status (single), familiarity (low), past mental health treatment in public service (no), family member treated in public mental health service (no), past hospitalization (no), national security insurance (no).

Table S4. Full model of predictors of 'Expanding rehabilitation and community services' as a second-ranked goal of mental healthcare vision, and 'organizational culture' as the second most reported challenge in technology implementation *among professionals*

|  | Expanding rehabilitation and community services | | | | Organizational culture as the third most reported challenge | | | |
| --- | --- | --- | --- | --- | --- | --- | --- | --- |
| *Demographic factors (Block 1)* | B | OR | 95% CI | *p* | B | OR | 95% CI | *p* |
| Gender | 0.21 | 1.23 | 0.65-2.32 | 0.51 | 0.14 | 1.15 | 0.60-2.23 | 0.67 |
| Age: 40-49 | 0.06 | 1.06 | 0.55-2.03 | 0.85 | -0.27 | 0.76 | 0.40-1.43 | 0.40 |
| Age: 50 and above | -0.38 | 0.68 | 0.36-1.27 | 0.23 | **-0.76** | **0.46** | **0.24-0.87** | **0.02** |
| Family status: Married/relationship | 0.33 | 1.38 | 0.55-3.46 | 0.48 | 0.33 | 1.40 | 0.54-3.62 | 0.48 |
| Family status: Separated/divorced | 0.13 | 1.13 | 0.30-4.19 | 0.84 | 0.00 | 1.00 | 0.24-4.13 | 0.99 |
| *Professional factors (Block 2)* |  |  |  |  |  |  |  |  |
| Psychologists and social workers | -0.25 | 0.77 | 0.36-1.60 | 0.49 | **0.91** | **2.48** | **1.11-5.55** | **0.03** |
| Art and occupational therapists, other | -0.21 | 0.80 | 0.36-1.77 | 0.59 | 0.53 | 1.71 | 0.71-4.10 | 0.22 |
| Years of experience: above 10 years | -0.60 | 0.54 | 0.27-1.10 | 0.09 | 0.03 | 1.03 | 0.51-2.08 | 0.92 |
| Working in the Public Service | 0.47 | 1.69 | 0.53-4.88 | 0.40 | -0.36 | 0.69 | 0.22-2.15 | 0.52 |
| High familiarity with public mental health | 0.24 | 1.28 | 0.75-2.18 | 0.36 | -0.85 | 0.59 | 0.34-1.01 | 0.06 |

*Notes.* Reference group for age (0-39 years), gender (male), family status (single), profession (psychiatrics), years of experience (under 10 years), working in public service (no), familiarly with public mental health (low and medium).

Table S5. Full model of predictors of 'Alternatives to psychiatric hospitalization' as third-ranked priority in future vision, and 'Improving accessibility to knowledge' as the second main technological need among *service users*

|  | Alternatives to psychiatric hospitalization | | | | Improving accessibility to knowledge | | | |
| --- | --- | --- | --- | --- | --- | --- | --- | --- |
| *Demographic factors (Block 1)* | B | OR | 95% CI | *p* | B | OR | 95% CI | *p* |
| Gender | 0.14 | 1.15 | 0.74-1.78 | 0.52 | -0.00 | 0.99 | 0.64-1.54 | 0.98 |
| Age: 30-39 | 0.06 | 1.07 | 0.61-1.86 | 0.82 | 0.28 | 1.33 | 0.76-2.31 | 0.31 |
| Age: 40 and above | 0.06 | 1.06 | 0.63-1.78 | 0.83 | 0.28 | 1.32 | 0.78-2.23 | 0.30 |
| Family status: Married/relationship | 0.15 | 1.16 | 0.74-1.80 | 0.51 | -0.12 | 0.89 | 0.56-1.40 | 0.61 |
| Family status: Separated/divorced | -0.12 | 0.89 | 0.49-1.62 | 0.70 | -0.39 | 0.67 | 0.36-1.25 | 0.21 |
| *Clinical factors (Block 2)* |  |  |  |  |  |  |  |  |
| Medium familiarity with public mental health | **0.83** | **2.31** | **1.20-4.44** | **0.01** | 0.42 | 1.53 | 0.82-2.87 | 0.18 |
| High familiarity with public mental health | **1.10** | **2.99** | **1.55-5.77** | **0.00** | **0.80** | **2.21** | **1.18-4.13** | **0.01** |
| Past treatment in public service | 0.30 | 1.35 | 0.82-2.20 | 0.23 | -0.12 | 0.88 | 0.54-1.44 | 0.62 |
| Family member treated in public service | -0.26 | 0.77 | 0.52-1.14 | 0.20 | -0.15 | 0.85 | 0.58-1.27 | 0.44 |
| Past psychiatric hospitalization | -0.88 | 0.41 | 0.23-0.75 | 0.00 | 0.46 | 1.58 | 0.90-2.81 | 0.11 |
| Social benefit/rehabilitation service | 0.50 | 1.64 | 0.90-2.99 | 0.10 | 0.05 | 1.05 | 0.60-1.87 | 0.85 |

*Notes.* Reference group for age (0-29 years), gender (male), family status (single), familiarity (low), past mental health treatment in public service (no), family member treated in public mental health service (no), past hospitalization (no), national security insurance (no).

Table S6. Full model of predictors of ' Improving treatment continuity' services as a third-ranked goal of mental healthcare vision, and 'Employee burnout' as the second most reported challenge in technology implementation *among professionals*

|  | Improving treatment continuity | | | | Employee burnout | | | |
| --- | --- | --- | --- | --- | --- | --- | --- | --- |
| *Demographic factors (Block 1)* | B | OR | 95% CI | *p* | B | OR | 95% CI | *p* |
| Gender | 0.10 | 1.11 | 0.56-2.21 | 0.77 | -0.04 | 0.95 | 0.49-1.85 | 0.89 |
| Age: 40-49 | **-0.74** | **0.47** | **0.24-0.93** | **0.03** | -0.47 | 0.62 | 0.31-1.23 | 0.17 |
| Age: 50 and above | -0.47 | 0.62 | 0.32-1.23 | 0.17 | 0.42 | 1.52 | 0.81-2.86 | 0.19 |
| Family status: Married/relationship | 0.65 | 1.92 | 0.74-4.95 | 0.18 | -0.20 | 0.81 | 0.32-2.07 | 0.67 |
| Family status: Separated/divorced | 0.33 | 1.38 | 0.37-5.27 | 0.62 | -0.17 | 0.84 | 0.22-3.20 | 0.80 |
| *Professional factors (Block 2)* |  |  |  |  |  |  |  |  |
| Psychologists and social workers | 0.46 | 1.51 | 0.72-3.17 | 0.27 | -0.43 | 0.64 | 0.31-1.34 | 0.24 |
| Art and occupational therapists, other | 0.39 | 1.33 | 0.60-2.96 | 0.47 | -0.25 | 0.78 | 0.35-1.72 | 0.54 |
| Years of experience: above 10 years | 0.17 | 1.18 | 0.56-2.34 | 0.63 | 0.23 | 1.26 | 0.62-2.57 | 0.52 |
| Working in the Public Service | 0.28 | 1.32 | 0.37-4.52 | 0.67 | 0.00 | 1.00 | 0.34-2.94 | 0.99 |
| High familiarity with public mental health | 0.44 | 1.56 | 0.88-2.75 | 0.12 | 0.23 | 1.26 | 0.73-2.21 | 0.41 |

*Notes.* Reference group for age (0-39 years), gender (male), family status (single), profession (psychiatrics), years of experience (under 10 years), working in public service (no), familiarly with public mental health (low and medium).
